# Infection by *Microsporum canis* in Paediatric Patients: A Veterinary Perspective

**DOI:** 10.3390/vetsci4030046

**Published:** 2017-09-19

**Authors:** Mario Pasquetti, Anna Rita Molinar Min, Stefania Scacchetti, Andrea Dogliero, Andrea Peano

**Affiliations:** 1 Dipartimento di Scienze Veterinarie, Università di Torino, 10095 Grugliasco, Italy; mario.pasquetti@unito.it (M.P.); annarita.molinar@unito.it (A.R.M.M); andrea.dogliero@unito.it (A.D.); 2 Private Practitioner (Veterinary), 12030 Cavallermaggiore (CN), Italy; stefy70vet@yahoo.it

**Keywords:** *Microsporum canis*, dermatophytes, ringworm, paediatric, cat

## Abstract

*Microsporum canis* is a dermatophyte fungus of which cats and dogs are recognized as the natural hosts. *M. canis* is also easily transmitted to humans, causing lesions to the glabrous skin (*tinea corporis*) and to the head (*tinea capitis*). The present study describes some cases of infection with *M. canis* in children from a veterinary perspective, highlighting some important features of this clinical entity (e.g., the necessity to identify the animal source of infection with appropriate diagnostic tests; the fact that infected cats may present with no or atypical dermatological signs; and the importance of the environment as a fungal reserve).

## 1. Introduction

*Microsporum canis* belongs to the group of dermatophyte fungi, which are closely related organisms that have the ability to invade the stratum corneum of the epidermis and keratinized tissues derived from it, such as skin, nails, and hair of humans and animals. These fungi produce an infection called dermatophytosis, commonly referred to as ringworm or *tinea* [1,2]. *M. canis* is the most common dermatophyte in cats and dogs, with cats considered to be the most important reservoir hosts. This organism has also been occasionally reported in a number of other domestic and wild animals [2,3,4,5]. *M. canis* is known to mainly reproduce asexually through a mitotic process, and the propagules that originate from the asexual reproduction (conidia) vary according to the context in which the fungus is located. During hair/scale invasion, hyphae are fragmented to produce masses of small arthroconidia, which represent the infective parts of the fungus [6]. In culture plates, fungal reproduction yields spindle-shaped macro-conidia and one-celled microconidia [2,6].

*M. canis* is widespread worldwide (particularly in Europe, the eastern Mediterranean, and South America) and plays an important zoonotic role. *M. canis* is a very frequent agent of *tinea capitis*, mainly in children, and can also cause highly inflamed lesions on glabrous skin (*tinea corporis* and *tinea faciei*) [1]. This study describes some cases of *M. canis* infection involving paediatric patients from a veterinary perspective.

## 2. Materials and Methods

**Figure 1:**
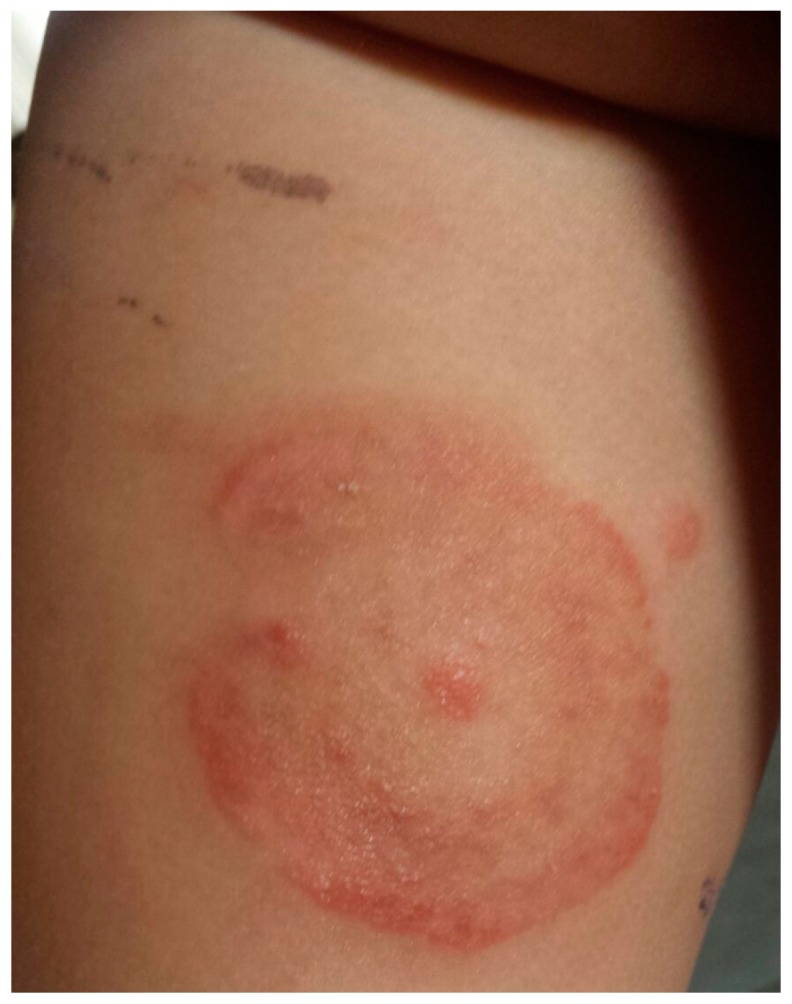
*Tinea corporis* due to *M. canis* in a child (case # 3).

The cases in this study involved animals taken for examination in two veterinary clinics in northern Italy (the veterinary clinic of the Department of Veterinary Sciences of Turin and a private veterinary clinic in the Cuneo province). The examination was requested because children in contact with the animals had developed *tinea corporis* due to *M. canis* (Figure 1).

**Figure 2:**
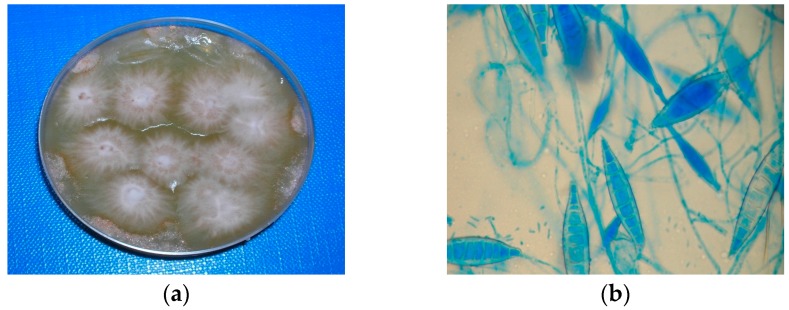
(**a**) Colonies of *M. canis* in culture; (**b**) Microscopic aspect, macro- and micro-conidia.

For each animal, a complete clinical examination was performed. The animals were sampled using the tooth-brush technique [6], where a toothbrush is combed over the entire hair coat to accumulate hair and keratin debris and is then pressed onto the surface of a culture plate. Contact plates (Rodac, PBI International S.P.A., Milano, Italy) were employed to evaluate the fungal contamination inside the homes of provenance. In the different rooms (kitchen, bathroom, living room, bedroom), a series of plates were pressed on floors, pieces of furniture (tables, sofa, beds, etc.), and various objects (curtains, carpets, towels, etc.). The growth medium used for the cultures was Mycobios Selective Agar (Biolife, Milano, Italy). Plates were incubated at 25 °C and examined daily for 2 weeks. Fungal colonies were identified to species level based on their morphology and microscopic features (Figure 2).

## 3. Results

### 3.1. Case 1 (April, 2014)

**Figure 3:**
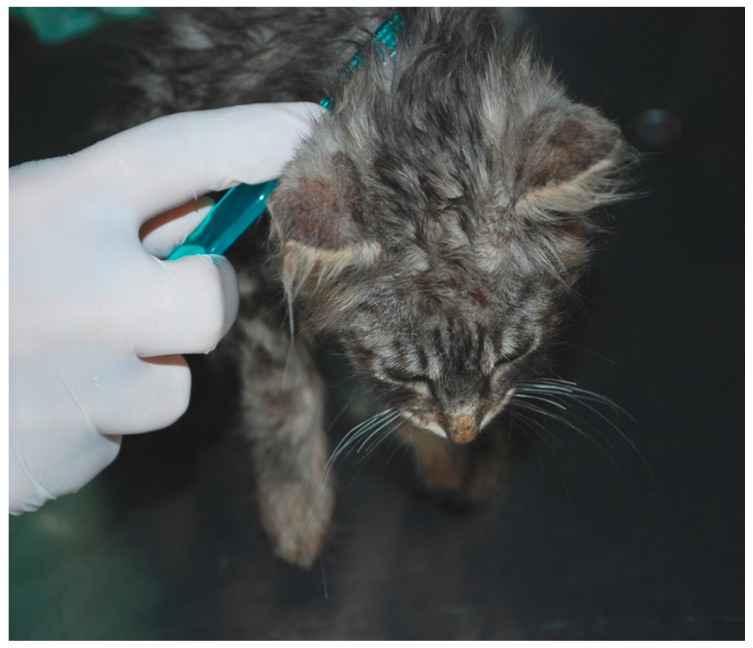
A kitten infected by *M. canis* (source of infection in case # 1) with no evidence of clinical lesions, sampled by the tooth-brush technique.

The source of infection (SOI) was demonstrated to be a stray kitten that had been adopted. The cat had no evidence of clinical lesions (Figure 3).

**Figure 4:**
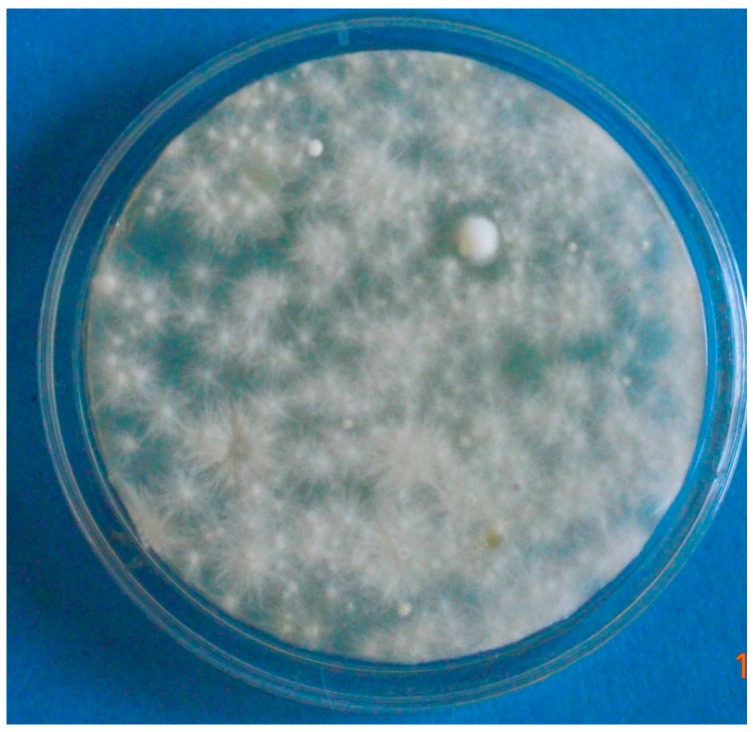
Environmental sample (contact plate from sofa, case # 1). Widespread growth of colonies of *M. canis.*

Heavy environmental contamination was also detected (Figure 4). 

The cat was successfully treated using itraconazole with oral therapy (Itrafungol^®^, Elanco Italia S.p.A., Sesto Fiorentino, Italy) and miconazole (Demicol^®^, Ceva Salute Animale S.p.A., Agrate Brianza, Italy) with topical therapy. Environmental decontamination was performed by vacuuming to remove infected hairs (with subsequent disinfection of the vacuum cleaner); washing of surfaces and objects with household bleach where possible (e.g., floors in the bathroom or in the kitchen) or enilconazole spray (Clinafarm, Elanco Italia S.p.A, Sesto Fiorentino, Italy); and disinfecting of washable textiles via mechanical washing. Contamination was monitored over time, and cleaning procedures were stopped when fungal culture results were negative.

### 3.2. Case 2 (March, 2016)

The SOI was a pure-breed cat (Sphynx) purchased from a breeder. The sole clinical abnormality was the presence of pigmented areas that disappeared after antifungal therapy (itraconazole + topical miconazole). Heavy environmental contamination was also demonstrated. Frequent vacuuming was recommended to remove gross debris, followed by cleaning of surfaces and objects using a peroxidisulfate formulation containing an anionic surfactant and organic acids (VI-SEPT Tablets, Medicaline) until culture results were negative. Washable textiles were disinfected via mechanical washing using the same product (VI-SEPT Tablets, Medicaline S.r.l, Monfalcone, Italy).

### 3.3. Case 3 (May, 2017)

**Figure 5:**
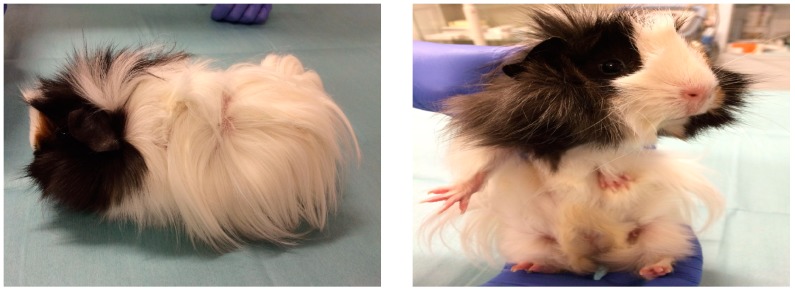
A guinea pig presented as the probable cause of infection by *M. canis* in a child (case # 3).

A guinea pig kept as a pet by the family was presented as the probable cause of infection by *M. canis*. The animal had no evidence of dermatological lesions (Figure 5).

However, fungal cultures from the animal and the environment were negative. The parents reported that 2 weeks previously the child had attended a birthday party in a park during which he had played with a stray cat.

## 4. Discussion

During recent years, the incidence of *M. canis* infection in humans has strongly increased in Europe [7,8]. In particular, this dermatophyte is now considered one of the most prevalent causes of *tinea capitis* in children [8]. Outbreak episodes of children presenting with *tinea corporis* have been described in different publications, some of which date back to several years ago [9,10,11,12].

The description of cases from a veterinary perspective is useful, as studies in the literature generally report the overall statistics of dermatological centres [7,8], while scarce attention is given to factors involving the animal source of the problem. However, this aspect—for example, the animal species involved, the age, breed, clinical presentation, etc.—is clearly important in order to define the risk factors for humans.

The findings of the present study indicate several important aspects of human infections by *M. canis*.

Case # 3 illustrates that the SOI is not necessarily represented by animals inhabiting with the infected person, and that the animal involvement must be proven by laboratory examinations. Specifically, the guinea pig hosted in the household was demonstrated not to have played a role in the episode of *M. canis* infection, which was probably due to a stray cat the child had been in contact with during a birthday party. In the present study, examination of the animal acting as the SOI by *M. canis* was carried out by fungal culture using the toothbrush technique. This is considered as the test of choice to demonstrate the presence of dermatophyte conidia on the animal hair-coat [6,13,14]. Other commonly used diagnostic tests include direct microscopic examination of the hair, which allows observation of the fungal elements (hyphae and arthroconidia) invading the hair shaft, and the Wood’s lamp examination [13,14]. This last test is considered a good screening method for dermatophytosis in dogs and cats. When exposed to the light of the lamp, hairs invaded by most strains of *M. canis* glow yellow-green. The fluorescence is due to tryptophan metabolites produced by some dermatophyte species, including *M. canis* [14]. False positive and false negative results occur (most commonly due to inadequate equipment, lack of magnification, lack of patient compliance, poor technique, or lack of operator training [13]); therefore, results from Wood’s lamp examination should always be confirmed by other tests (direct examination and culture) [14].

Cases # 1 and 2 confirmed that cats are a frequent SOI, especially in children [10,11], although dogs (and occasionally other animals) may also be responsible [9]. 

Another important observation is that cats infected with *M. canis* may present with poor evidence of lesions (see case # 1) or atypical lesions (see case # 2). This illustrates the importance of always performing laboratory examinations to rule out the presence of *M. canis* in animals *prior to* their adoption, regardless of the presence of (typical) dermatological signs.

The role of the environment as a reserve of fungal elements is another notable aspect. Arthroconidia may persist for months or years, particularly when embedded in hair or skin scales, on floors, walls, and objects associated with grooming, transportation, and housing of animals (cages, combs, brushes, clippers, blankets, etc.) [2]. Sofas, beds, chairs, and furniture can be contaminated as well [13], as confirmed in our study. Although the contact with a contaminated environment alone in the absence of infected animals is considered a rare source of infection in both people and animals [13], decontamination is important to minimize the risk of re-infection and to reduce the treatment time required by the animals [14]. Before the application of any cleaning product, it is important to remove all debris by vacuuming or sweeping, since disinfectants do not work in the presence of organic material [13,14]. Products for disinfection should be nontoxic with a low irritancy to the animals and users, and they should be compatible with the surfaces that they are applied to [13].

Examples of effective commercially-available products are household bleach at concentrations ranging from 1:10 to 1:100; enilconazole (in solution or as a smoke fumigant formulation); accelerated hydrogen peroxide (AHP); and potassium peroxymonosulfate [13,14]. Notably, the use of most of these products in a household context requests some caution. For example, bleach has unpleasant odour, can cause damage to hard surfaces, and can result in discoloration of fibres and coloured surfaces, as well as damage to floor finishes. Moreover, it is an irritant [13]. Enilconazole formulations are considered very effective [13], but they are only licensed for use in farm buildings (poultry and rabbit breeding). The product used in case # 2 (VI-SEPT Tablets, Medicaline) is instead licensed for domestic use, and can thus be employed—without problems—for disinfecting surfaces as well as objects and textiles.

The treatment of infected animals until mycological cure is obtained is obviously essential to prevent re-infection for humans that they are in contact with. The cats in the present study were treated following a commonly accepted protocol (a combination of systemic and topical therapy based on azole compounds) [13,14]. In recently published guidelines [13], the possibility of using antifungal vaccines in the case of feline dermatophytosis has also been discussed. In particular it is stated that, in light of the existing literature, antifungal vaccines do not protect against challenge exposure to dermatophyte infection, but may represent a useful adjunct therapy [13]. Though this evaluation is encouraging, and some vaccines against *M. canis* are commercially available in certain countries, vaccination is scarcely employed in everyday clinical practice.

Finally, the cases described show that in the case of human infections, an animal is immediately deemed responsible as the SOI. However, outbreaks without animal presence have also been reported in schools [11,12] and in a nursery [15]. This shows that although it is considered rare and self-limiting, human-to-human transfer of *M. canis* can occasionally occur.

## 5. Conclusions

During the course of human infection by *M. canis*, veterinary intervention is crucial to precisely identify the SOI, which in turn avoids the risk of re-infection.
